# Enterovirus Replication Organelles and Inhibitors of Their Formation

**DOI:** 10.3389/fmicb.2020.01817

**Published:** 2020-08-20

**Authors:** Xinhong Li, Mingshu Wang, Anchun Cheng, Xingjian Wen, Xumin Ou, Sai Mao, Qun Gao, Di Sun, Renyong Jia, Qiao Yang, Ying Wu, Dekang Zhu, Xinxin Zhao, Shun Chen, Mafeng Liu, Shaqiu Zhang, Yunya Liu, Yanling Yu, Ling Zhang, Bin Tian, Leichang Pan, Xiaoyue Chen

**Affiliations:** ^1^Institute of Preventive Veterinary Medicine, Sichuan Agricultural University, Chengdu, China; ^2^Key Laboratory of Animal Disease and Human Health of Sichuan Province, Sichuan Agricultural University, Chengdu, China; ^3^Avian Disease Research Center, College of Veterinary Medicine, Sichuan Agricultural University, Chengdu, China

**Keywords:** enteroviruses, replication organelles, biogenesis, lipid metabolism, inhibitors

## Abstract

Enteroviral replication reorganizes the cellular membrane. Upon infection, viral proteins and hijacked host factors generate unique structures called replication organelles (ROs) to replicate their viral genomes. ROs promote efficient viral genome replication, coordinate the steps of the viral replication cycle, and protect viral RNA from host immune responses. More recent researches have focused on the ultrastructure structures, formation mechanism, and functions in the virus life cycle of ROs. Dynamic model of enterovirus ROs structure is proposed, and the secretory pathway, the autophagy pathway, and lipid metabolism are found to be associated in the formation of ROs. With deeper understanding of ROs, some compounds have been found to show inhibitory effects on viral replication by targeting key proteins in the process of ROs formation. Here, we review the recent findings concerning the role, morphology, biogenesis, formation mechanism, and inhibitors of enterovirus ROs.

## Introduction

Enteroviruses, members of the *Picornaviridae* family *Enterovirus* genus, include poliovirus (PV), enterovirus (EV), coxsackievirus (CV), and human rhinovirus (HRV), which can cause poliomyelitis, meningitis, hand-foot-and-mouth disease, respiratory disease, and so on (Tapparel et al., 2013). Enteroviruses are non-enveloped particles, the capsid is a symmetric icosahedral capsid, and the genome is non-segmented single-stranded positive-strand RNA (about 7.5 kb). From the 5’end, the first element of the genome is VPg (3B), which is connected to the genome by covalent bond. After the VPg is the 5’non-translated region (NTR), where the “cloverleaf” and the internal ribosome entry site (IRES) are. This is followed by a single large open reading frame (ORF). The 3’end is a short 3’NTR, containing a poly (A) tail (Jiang et al., 2014; Peersen, 2017). The ORF encodes a polyprotein, about 2,300 amino acids. It is artificially separated into three sections, P1, P2, and P3. P1 encodes structural proteins, which are related to the assembly of viral capsids. P2 and P3 encode non-structural proteins and participate in virus replication and virus-host interaction (Belov et al., 2012; Cao et al., 2012; Wen et al., 2015).

Positive-strand RNA viruses from different families (such as *Picornaviridae, Flaviviridae*, *Coronaviridae*, and *Togaviridae*) rely on host cell machinery to replicate their genome and generate progeny viral particles. Upon infection, virus induce the rearrangements of cellular membranes to form specific intracellular compartment called virus factory or viroplasma, which contain unique platforms known as replication organelles (ROs) to replicate viral RNA (vRNA). Virus replication complexes include viral proteins needed for viral replication and coopted host factors, which constitute ROs with the sites of virus particle assemble (Novoa et al., 2005; Owino and Chu, 2019; Sachse et al., 2019). Like all positive-strand RNA viruses, enterovirus infection causes the cellular membranes modified to produce ROs for replication of vRNA. After years of efforts by scientists, the morphology, biogenesis, and related pathways of ROs biogenesis (including the secretory pathway, autophagy, and lipid metabolism) are discovered. In addition, finding potential targets for antiviral therapies during the ROs formation process is a very active field of research. In this review, we outline the research progress about the role, morphology, and biogenesis of enterovirus ROs, and briefly describe inhibitors that target ROs formation. It improves our understanding of enterovirus replication and provides reference for the development of anti-enterovirus drugs.

## Function of RO

It is theorized that ROs promote virus replication by raising the concentrations of proteins involved in virus replication, providing platform to assemble multisubunit ROs, and effectively coordinating the different steps of the replication process to improve replication efficiency (McCloskey and Poo, 1986; Ravindran et al., 2016). The membrane lipids on the ROs can regulate virus polyprotein processing and enzyme activity. For example, cholesterol on ROs is vital for regulating the processing kinetics of viral 3CD^pro^ (Ilnytska et al., 2013). ROs also help viruses evade innate immunity by hiding double-stranded RNA (dsRNA), a replication intermediate of vRNA, from cellular sensors (den Boon and Ahlquist, 2010; Belov et al., 2012; Viktorova et al., 2018). In the case of PV, membranous scaffold of the ROs is essential to protect vRNA from innate immunity, especially in multicycle replication conditions (Viktorova et al., 2018). However, innate immunity do not increase when CVB3 ROs generation is delayed, suggesting that ROs are not indispensable for innate immune evasion (Melia et al., 2017).

## The Morphology and Biogenesis of RO

Although the RO structures induced by different viruses have their own unique characteristics, their structures are very similar, suggesting that viruses have adopted a conserved mechanism to assemble ROs during long-term evolution (Zhang et al., 2015). There are two forms of ROs. The cell membrane of the ER or other donor organelles recess inward, forming a concealed structure containing all the elements necessary for replication (formed by negative curved membranes), which is adopted by dengue virus, Zika virus, and so on. Other viruses (hepatitis C virus, picornaviruses, arterivirus, and coronavirus) produce double-membrane vesicles (DMVs), which are formed by squeezing the donor membrane (formed by positive curved membranes). Enteroviruses induce membrane rearrangement by the second way (Bienz et al., 1992; Paul and Bartenschlager, 2013; Roulin et al., 2014; Blanchard and Roingeard, 2015; Ertel et al., 2017; Strating and van Kuppeveld, 2017). Viral proteins participate in the formation of ROs. PV 2BC and 3A proteins cause ER change, triggering the arising of ROs (Suhy et al., 2000). EV71 2C protein interacts with reticulon 3 (RTN3), which can change the curvature of the ER membrane (Tang et al., 2007).

**FIGURE 1:**
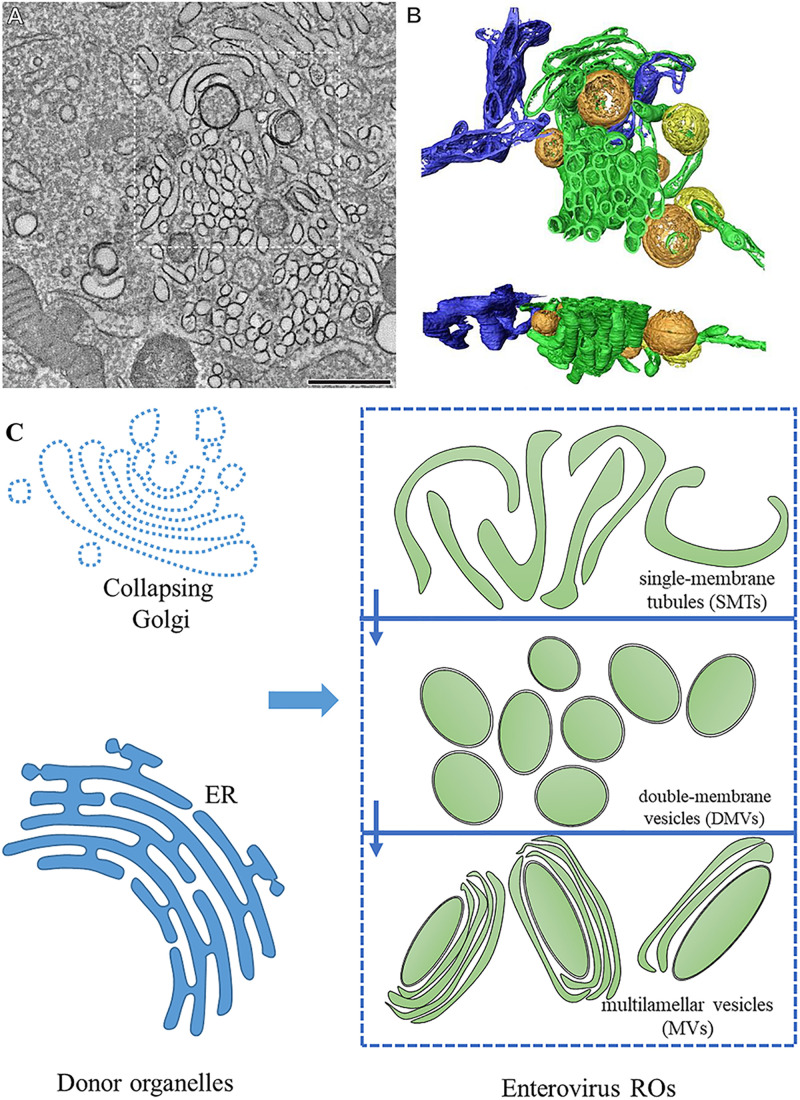
The morphology of enterovirus ROs. **(A)** Tomographic slice through the serial tomogram of a CVB3-infected cell at 5 h post-infection, with clusters of SMT and sparsely embedded DMVs. Scale bar is 500 nm. **(B)** Top and side views of a surface-rendered model of the region boxed in panel **(A)** showing SMT (green), open DMVs (orange), closed DMVs (yellow), and ER (blue). **(C)** Pattern about of RO morphology. Enterovirus ROs are generated from the ER and the Golgi. During the early stages of infection, enteroviruses produce ROs with single-membrane tubule (SMT) morphology. SMTs transform into double-membrane vesicles (DMVs) and multilamellar vesicles (MVs) with the progression of infection. **(A,B)** Adapted from Limpens et al. (2011). The original images have been published under the Creative Commons Attribution-Non-commercial-Share Alike 3.0 Unported license. We have obtained author’s permission.

For a long time, in the studies using two-dimensional electron microscopy, enterovirus ROs were regarded as vesicles with single or double membranes (Kallman et al., 1958; Bienz et al., 1980; Egger et al., 1996; Paul and Bartenschlager, 2013; Romero-Brey and Bartenschlager, 2014). In recent years, the three-dimensional structure of enteroviral ROs has been revealed. During infection, the structures of ROs are constantly changing, indicating that ROs are dynamic. They seem to evolve from each other with the process of viral infection. In early infection, enteroviruses ROs appear in cytoplasm with the form of single-membrane tubules (SMTs). With the progress of infection, SMTs transform into DMVs via membrane pairing and fission. In the late stage of infection, multilamellar vesicles (MVs) appear in the cytoplasm. The formation of DMVs and MVs is via membrane apposition, enwrapping, and fusion (Limpens et al., 2011; Belov et al., 2012; van der Schaar et al., 2016b; Oh et al., 2018; Wolff et al., 2020). DMVs are enwrapped by one or multiple cisternae to form MVs (Limpens et al., 2011). The membrane structures formed after enterovirus infection are discontinuous, and SMT and DMV are independent, separate structures (Figure 1). Enterovirus ROs are different from ROs of encephalomyocarditis virus, equine arteritis virus, and severe acute respiratory syndrome (SARS) coronavirus, which induce interconnected DMVs to comprise reticulovesicular networks (Knoops et al., 2008, 2012; Melia et al., 2018). Enterovirus ROs apparently lack of pores which let the vRNA enter ROs inside, suggesting vRNA replication takes place on the enterovirus ROs cytoplasmic leaflet (Limpens et al., 2011; Belov et al., 2012; Strating and van Kuppeveld, 2017).

Do the three structures (SMTs, DMVs, and MVs) all participate in genome replication? Researches on PV and CVB3 infection elucidated that there is a link between viral proteins, dsRNA, and both SMTs and DMVs. In the exponential phase of vRNA replication, the structure of ROs mainly forms SMTs, suggesting that the early and mid-term SMTs are related to the onset and most dense rates of RNA synthesis. DMVs appear in large numbers after the most active stage of vRNA synthesis, indicating that DMVs may contribute to other steps of viral life cycle, such as virus assembly (Limpens et al., 2011; Belov et al., 2012; Oh et al., 2018). This is different from HCV that DMVs of HCV is the place vRNA replication take place (Lee et al., 2019). DMVs of PV are postulated to mediate non-lytic release of progeny virus particles (Kirkegaard and Jackson, 2005; Chen et al., 2015). DMVs of SARS-coronavirus are suggested to conceal vRNA to escape the antiviral response triggered by dsRNA (Knoops et al., 2008).

Previous studies have shown that enteroviruses form ROs that are juxtaposed with exit sites of the ER, where viruses anchor the viral replication mechanism (Suhy et al., 2000; Hsu et al., 2010; Limpens et al., 2011). During the process of enteroviral infection, Golgi collapse and cannot be detected, suggesting that the membranes of the ROs may be derived from the Golgi (Hsu et al., 2010; Mousnier et al., 2014; van der Schaar et al., 2016b). The origin of CVB3 ROs was elucidated. Biogenesis of CVB3 ROs was found to occur first at the ER and then at Golgi membranes (Melia et al., 2019). In the early infection, when the level of viral proteins is low, PV and CVB3 make use of the Golgi compartment and *trans-*Golgi network (TGN) to synthesize vRNA (Hsu et al., 2010; van der Schaar et al., 2016b). The CVB3 3A-H57Y mutant, which can replicate under phosphatidylinositol 4-kinase beta (PI4KB) inhibition and can not form ROs, replicates at the Golgi (Melia et al., 2017).

## Role of the Secretory Pathway in RO Biogenesis

Several studies have investigated whether the replication of PV and CVB3 requires guanine nucleotide exchange factor 1 (GBF1), a guanine nucleotide exchange factor (GEF) of ADP ribosylation factor (Arf), involved in the secretory pathway. GBF1 recycles Arf from GDP-bound form to GTP-bound form (Belov et al., 2007a; Lanke et al., 2009). The replication of EV71 and PV depends on GBF1-mediated activation of Arf (Wang et al., 2014; Viktorova et al., 2019). Upon CVB3 infection, newly synthesized proteins, such as 3A protein, are located on secretory organelle membranes and recruit effector through GBF1/Arf1, thereby enhancing the accumulation of PI4KB over Coat protein complex I (COPI), causing the producing of uncoated phosphatidylinositol 4-phosphate (PI4P)-enriched structures adjacent to ER exit sites (Hsu et al., 2010). PV requires a little subset of normal GBF1 functions. GBF1 components are crucial to support virus replication under different situations. 3A-GBF1 interaction and membrane targeting function are mediated by the GBF1 C-terminal domain, which are vital to recruiting GBF1 to ROs. If either is damaged, functional ROs are still formed, but when both are inactivated, no RO is formed (Viktorova et al., 2019). Viral protein 3CD has also been demonstrated to be related to the GBF1, Arf1, and PI4KB pathways (Belov et al., 2007b; Banerjee et al., 2018). Other GEFs (BIG1 and BIG2) are also involved in the replication of enteroviruses. After PV infection, viral 3CD protein promotes the binding of Arf to the membrane by recruiting BIG1 and BIG2, which is linked to the ROs formation (Belov et al., 2007a, b).

COPI and Coat protein complex II (COPII) vesicles are important part of the transport between ER and Golgi. COPI vesicles assume the role of transporting goods to Golgi, limiting lipid storage and maintaining lipid homeostasis. COPII act a vital role in the transport of proteins and lipids from ER to Golgi (Lee et al., 2004; Beller et al., 2008). COPI and COPII both are involved in the formation of enterovirus RO. Brefeldin A (BFA), an inhibitor that targets COPI, shows a strong inhibitory action on the vRNA replication of PV. EV11, CVA-21, and CVB3 replication is also inhibited by BFA, indicating that COPI vesicle trafficking is instrumental to the replication of enteroviruses (Irurzun et al., 1992; Maynell et al., 1992; Gazina et al., 2002; van der Linden et al., 2010). Replication of EV-A71 requires COPI, which may be recruited to ROs through the interaction of the COPI coatomer and 2C protein of EV-A71 (Wang et al., 2012). The generation of PV ROs is associated in cellular COPII germination mechanism (Rust et al., 2001; Trahey et al., 2012).

UDP-glucose glycoprotein glucosyltransferase 1 (UGGT1) is a crucial protein in ER involved in the unfolded protein response (UPR). UGGT1 and EV-A71 3D polymerase (3D^pol^) coprecipitate with other factors related to ROs to encourage EV-A71 replication (Huang et al., 2017; Table 1).

**TABLE 1:** The host proteins associated with RO biogenesis.

Host protein	Virus (related viral protein)	Function in RO biogenesis	References
GBF1/Arf1	EV71 PV(3CD) CVB3(3A)	Cooperate viral 3A to recruit PI4KB or participate in ROs formation	Belov et al., 2007b; Hsu et al., 2010
BIG1 BIG2	PV(3CD)	Linked to the ROs formation	Belov et al., 2007a, b
COPI	PV EV11 CVA-21 CVB3 EV-A71(2C)	Related to ROs formation	Maynell et al., 1992; Gazina et al., 2002; van der Linden et al., 2010
COPII	PV	COPII germination mechanism is related to ROs formation	Rust et al., 2001
UGGT1	EV-A71(3D)	UGGT1 and 3D^pol^ coprecipitate with other factors related to ROs to encourage EVA71 replication	Huang et al., 2017
SNAP29	CVB3(3C) EV-D68(3C)	Promote autophagosomes accumulation to provide additional membrane scaffolds for ROs generation	Mohamud et al., 2018
PLEKHM1	CVB3(3C)		
BPIFB3	CVB	Related to ROs morphology	Delorme-Axford et al., 2014
FASN	PV CVB3	Synthesize FAs on ROs to synthesize PC	Rassmann et al., 2007
ACSL3	PV(2A)	Induce increased import of FAs in infected cells and up-regulation of phospholipid synthesis	Nchoutmboube et al., 2013
CCTα	PV(2A)	Bind to the ROs membrane to synthesize PC	Viktorova et al., 2018
ACBD3	Enterovirus(3A)	Cooperate viral 3A to recruit PI4KB	Greninger et al., 2012; Lei et al., 2017; Lyoo et al., 2019
PI4KB	Enterovirus(3A)	Phosphorylate PI to PI4P	Hsu et al., 2010; Spickler et al., 2013; Roulin et al., 2014
c10orf76	CVA10 PV	Contribute to proper Arf1 activation and increases the PI4P level of ROs	McPhail et al., 2020; Voilquin et al., 2020
HSL	HRV-A16	Hydrolyze cholesterol-ester stored in LDs to increase the cholesterol content of ROs	Roulin et al., 2014
OSBPL	HRV-A1A	Increase RO cholesterol content	Roulin et al., 2014
Rab11	PV(2BC) CVB3(2BC)	Related to transporting cholesterol to ROs and preventing cholesterol back to the PM	Hsu et al., 2010; Ilnytska et al., 2013
OSBP	HRV PV CVB3	Drive PI4P-cholesterol countercurrents to increase the level of cholesterol in ROs	Arita, 2014; Roulin et al., 2014

## Role of Autophagy in RO Biogenesis

Autophagy is a highly conservative lysosomal pathway unique to eukaryotic cells that degrades misfolded or redundant proteins in cells, damaged organelles, and intracellular pathogens. Autophagy is initiated by the generation of crescent isolation membrane vesicles called isolation membranes or phagophores. Subsequently, phagophores grow and fuse to form closed double-layer membrane autophagosomes. After elongation, autophagosomes have two directions. One is to form autolysosomes by fusing with lysosomes. The other is to fuse with late endosomes first to generate amphiphiles, and then fuse with lysosomes to produce autolysosomes (Lai et al., 2016; Pleet et al., 2018; Sun et al., 2019).

Multiple studies have reported that enteroviruses can induce and exploit autophagy to support the replication of virus. Studies find that the vesicles formed after enterovirus infection showed the features of autophagosomes, for example, the double-layer membrane structure of vesicles, the increase of esterified LC3, and the enrichment of cells with LC3 punctate location (Taylor and Kirkegaard, 2007; Wong et al., 2008; Yoon et al., 2008; Klein and Jackson, 2011; Alirezaei et al., 2012; Shi et al., 2015). Viral components of ROs (2B, 2BC, 3A, and 3AB) can trigger generation of esterified LC3 and/or double-membraned liposomes (Suhy et al., 2000; Wang et al., 2013; Wu et al., 2016; Lai et al., 2017). Induction of autophagy by rapamycin and nutrient deprivation leads to an increase in enteroviral replication. Contrary to the inhibition of autophagosomes formation by 3-methyladenine (3-MA), which can inhibit the formation of autophagosomes by inhibiting the activation of type III PI-3 kinase, and knockdown/knockout the genes vital for autophagosomes formation (ATG5, ATG7, ATG9, Beclin-1, VPS34, LC3, ULK1, and FIP2000) significantly reduced enterovirus replication (Sugden et al., 2005; Wong et al., 2008; Alirezaei et al., 2012; Abernathy et al., 2019). Using an antibody against dsRNA, dsRNA was found to colocalize with LC3 in late-infection of PV (Richards et al., 2014). The above results indicate that autophagy can promote virus replication and that autophagy may be involved in the ROs generation. Enteroviruses may use the autophagy pathway to provide membranous scaffolds for ROs generation after infection. CVB3 infection cause inhibition of the fusion of autophagosomes and lysosomes/late endosomes through CVB3 proteinase 3C cleave synaptosomal-associated protein 29 (SNAP29) and pleckstrin homology domain containing protein family member 1 (PLEKHM1), two vital proteins related to the autophagosomes fusion. This promotes autophagosomes accumulation to provide additional membrane scaffolds for ROs generation (Mohamud et al., 2018). SNAP29 was also found to be cleaved after EV-D68 infection (Corona et al., 2018). Bactericidal/permeability-increasing protein (BPI) fold-containing family B, member 3 (BPIFB3) was identified to be involved in CVB infection for it negatively regulated non-canonical form of autophagy. After silencing BPIFB3, CVB replication increased and CVB induced ROs morphology changed dramatically (Delorme-Axford et al., 2014; Table 1).

Other studies have shown that the autophagy pathway is associated with other aspects of the enterovirus life cycle, including entry, genome package, assembly, maturation, and release (Richards and Jackson, 2012; Chiramel et al., 2013; Mohamud and Luo, 2019).

## Lipid Metabolism of RO Biogenesis

The membrane is mainly composed of 5 kinds of phospholipids: phosphatidylcholine (PC), phosphatidylethanolamine (PE), phosphatidylserine (PS), phosphatidylinositol (PI), and sphingomyelin (SM) (Spector and Yorek, 1985). Phospholipids are amphiphilic and are divided into hydrophilic groups (head) and hydrophobic groups (tail) (De Craene et al., 2017). The head group is the main determinant of lipid properties (charge, shape, and interaction with proteins), and changes in fatty acids (FAs) length and saturation that make up the hydrophobic part of phospholipids also affect membrane properties (such as lipid accumulation and fluidity) (van Meer et al., 2008). Sterols are essential elements of membrane lipids and participate in regulating membrane fluidity, stability, and permeability. Cholesterol is the main sterol in mammalian cell membranes (De Craene et al., 2017). Compared with the organelle membrane in uninfected cells, the composition of enterovirus ROs is special. The ROs membrane is rich in PI4P, PC, and cholesterol (Ilnytska et al., 2013; Nchoutmboube et al., 2015; Altan-Bonnet, 2017).

### PC

During PV infection, the level of PC, the most common glycerophospholipid, increases (Vance et al., 1980; Zhang et al., 2016). The enrichment of PC with short palmitoylation likely produces more fluid membranes with intrinsic ability, thus forming convoluted tubular matrix of ROs (Belov, 2014).

**FIGURE 2:**
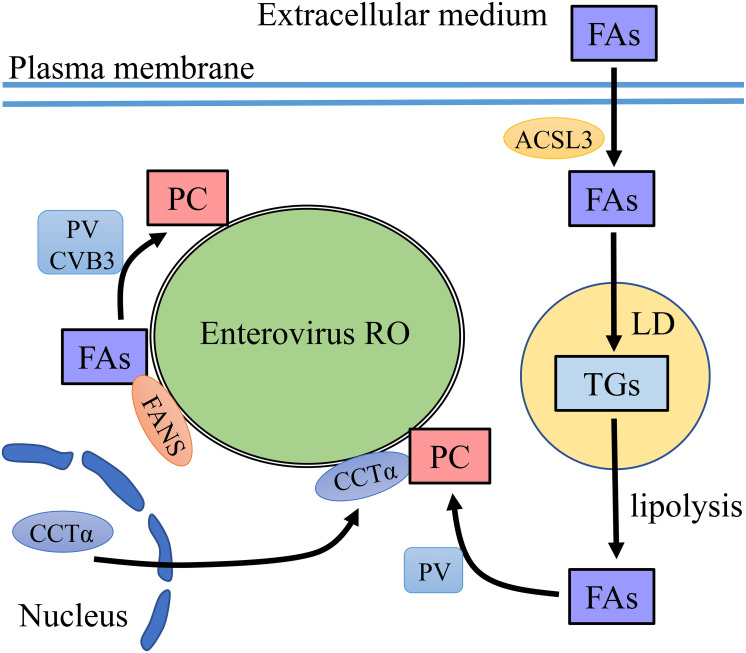
PC recruitment on enterovirus ROs. FAs are precursors for PC synthesis. Enteroviruses increase the level of FAs via two ways. One is to recruit FANS to enterovirus RO to synthesize FAs and then FAs convert to PC on the membrane of ROs. The other is to uptake FAs from the extracellular medium which depends on ACSL3. The imported FAs are targeted to TG synthesis and storage in LDs. FAs are released from LDs by lipolysis. CCTα translocates from the nucleus to the membrane of ROs, where CCTα synthesizes PC using FAs released from LDs.

To accumulate PC at ROs, it is vital to accumulate FAs to synthesize PC. One way is to recruit fatty acid synthase (FASN) to the ROs (Rassmann et al., 2007; Belov and van Kuppeveld, 2012). As the *de novo* synthesis pathway of FAs is needed, treatment with phospholipid synthesis inhibitors greatly reduced the replication and infectivity of PV and CVB3 (Guinea and Carrasco, 1990; Molla et al., 1993; Rassmann et al., 2007; Wilsky et al., 2012). Another way is to uptake FAs from extracellular medium (Nchoutmboube et al., 2013; Belov, 2014). Infection with PV activated the import of long-chain FAs, which related to the upregulation of cellular long-chain acyl-CoA synthetase long-chain family member 3 (ACSL3) activity. Viral protein 2A was required in this process independent of its protease activity. Imported FAs were converted into hydrophilic acyl-CoAs and synthesize triglycerides (TGs) stored in lipid droplets (LDs) in uninfected cells, while all newly imported FAs were used for PC biosynthesis in virus-infected cells (Nchoutmboube et al., 2013). However, a recent paper pointed out that upon PV infection, FAs taken from extracellular medium were not directly used for PC synthesis but were used to generate TGs stored in LDs (Laufman et al., 2019). Lipophagy and lipolysis are two ways to mobilize LD lipid. The lipids in LDs are transported to lysosomes by autophagy, called lipophagy. Lipases are recruited to LD surface to sequential hydrolysis of TGs stored within LDs, called Lipolysis (Zechner et al., 2017). After enterovirus infection, TGs within LDs transformed to FAs through lipolytic. Free FA was used to synthesize PC for ROs generation (Belov and van Kuppeveld, 2019; Laufman et al., 2019). PV infection induced massive translocation of CTP-phosphocholine-cytidyl transferase alpha (CCTα), the key enzyme in PC synthesis. CCTα transferred from the nuclei to the cytoplasm and this process was related to the protease activity of the 2A protein. CCTα bound to the ROs membrane to synthesize PC (Viktorova et al., 2018; Table 1; Figure 2).

### PI

Although PI accounts for only a small part of the total amount of phospholipids, it is the main determinant of organelle characteristics and functions. The most abundant PI in human cells is PI4P, which accounts for approximately 45% of the total intracellular PI. PI4P regulates membrane transport and metabolism by regulating the recruitment of host factors involved in lipid transfer (Payrastre et al., 2001; van der Schaar et al., 2016a; De Craene et al., 2017). PI4P is found to be related to enterovirus replication, such as PV, CVB3, HRV, and echovirus (Hsu et al., 2010; Altan-Bonnet and Balla, 2012; Ilnytska et al., 2013; Roulin et al., 2014). In mammalian cells, there are four enzymes that can phosphorylate PI to PI4P: phosphatidylinositol 4-kinase type 2 alpha (PI4K2A), phosphatidylinositol 4-kinase type 2 beta (PI4K2B), phosphatidylinositol 4-kinase alpha (PI4KA), and PI4KB (Delang et al., 2012; Dornan et al., 2016; Viaud et al., 2016; Burke, 2018). Upon enterovirus infection, ROs relied on PI4KB to phosphorylate PI to PI4P, creating a PI4P-enriched environment (Hsu et al., 2010; Spickler et al., 2013; Roulin et al., 2014). PI4P is suggested to be involved in the RNA polymerase complex and other host proteins involved in vRNA replication recruitment and stabilization on ROs membranes to promote the synthesis of vRNA (Hsu et al., 2010; Altan-Bonnet, 2017; Melia et al., 2017). In addition, it is involved in cellular lipid rearrangement, especially by recruiting oxysterol binding protein (OSBP) to transport cholesterol to ROs (Arita, 2014; Roulin et al., 2014).

Previous studies have demonstrated that enteroviral protein 3A can recruit and activate PI4KB. Although the 3A protein can coprecipitate with PI4KB, it does not show a direct interaction, indicating that the viral 3A protein recruits PI4KB through indirect interaction (Dorobantu et al., 2014). Thus far, two host factors have been found to participate in this process. One is GBF1. The viral 3A protein binds and regulates GBF1/Arf1 to increase the accumulation of PI4KB on ROs which catalyzes the production of PI4P (Hsu et al., 2010; Sasvari and Nagy, 2010). The second is the Golgi resident protein acyl-CoA-binding domain-containing 3 (ACBD3), also referred to as GCP60 and PAP7 (Greninger et al., 2012; Lei et al., 2017; Lyoo et al., 2019; Yue et al., 2019). ACBD3 is composed of an acyl-CoA binding (ACB) domain, a charged-amino-acid region (CAR), a glutamine-rich (Q) domain, and a Golgi dynamics (GOLD) domain (Yue et al., 2019). Enterovirus protein 3A binds to ACBD3 and then recruits PI4KB to ROs, where PI4KB produces a PI4P-enriched environment (Sasaki et al., 2012; Teoule et al., 2013; Lei et al., 2017; Xiao et al., 2017; Lyoo et al., 2019). The GOLD domain interacts with the enterovirus 3A protein, and the Q domain interacts with PI4KB (Klima et al., 2016; Chalupska et al., 2019). ACBD3 not only is an intermediary regulator of 3A and PI4KB but also participates in the correct localization of viral 3A protein (Lyoo et al., 2019). Crystal structures of the enterovirus 3A protein and ACBD3 complex have been characterized. Membrane bound protein 3A localizes on the membrane and binds to the GOLD domain of ACBD3 to form 3A-ACBD3 complex. Two 3A-ACBD3 complexes are close to each other, and a hydrophobic core is formed in the area near the four 3A N-terminal alpha helices to form a heterotetramer. The Q domain of ACBD3 binds to the N-terminus of PI4KB to mediate the direct, high-affinity interface between ACBD3 and PI4KB. 3A-ACBD3-PI4KB complex facilitates enterovirus replication (Horova et al., 2019; Smola et al., 2020). However, PI4KB and ACBD3 recruitment by CVB3 and HRV 3A likely independent of GBF1/Arf1 and ACBD3 (Dorobantu et al., 2014, 2015). And recent research about PI4KB using *trans* complementation with PI4KB mutants in a PI4KB-knockout cell line shows that interaction of PI4KB with host proteins (ACBD3, RAB11A, and 14-3-3) is not essential for EV replication once PI4KB has been expressed and that PI4KB is functionally independent from host proteins regarding EV replication (Arita, 2019). They are different from the above results, which may be due to the use of siRNA to knock down endogenous GBF1 and ACBD3 levels but not cause a complete knock out. Recruiting PI4KB in enterovirus replication requires only a small amount of GBF1/Arf1 and ACBD3 to function in ROs (Lyoo et al., 2019). Interestingly, reducing the content of endogenous ACBD3 by knock down or knock out increases the replication of HRV16, indicating that ACBD3 is a crucial factor in HRV16 replication (Xiao et al., 2017). PV 2BC protein and HRV 2B protein can also recruit PI4KB to RO membranes (Arita, 2014; Roulin et al., 2018).

**FIGURE 3:**
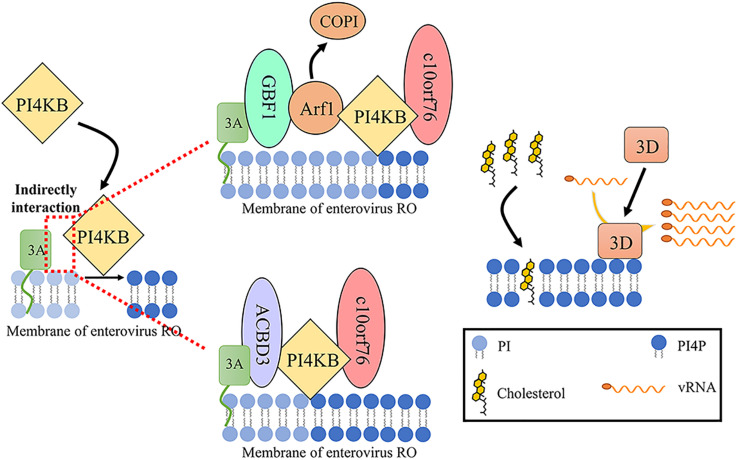
PI4P generation of enterovirus ROs. Enterovirus 3A recruits PI4KB to RO membrane through indirect interaction with PI4KB to phosphorylate PI to PI4P. Two intermediate factors have been found to be related to this process. One is GBF1. The viral 3A protein binds and regulates GBF1/Arf1, increasing the accumulation of PI4KB over COPI on RO. The other is ACBD3. ACBD3 not only is an intermediary regulator of 3A and PI4KB but also participates in the correct localization of viral 3A protein in the nucleus. PI4KB can recruit c10orf76 to the c10orf76-dependent virus RO membrane via the c10orf76-PI4KB interface. c10orf76 on the ROs may contribute to proper Arf1 activation and increases the PI4P level of ROs. PI4P is related to transporting cholesterol to ROs and recruiting 3D to generate vRNA on ROs (Sasvari and Nagy, 2010; McPhail et al., 2020).

Recently, c10orf76 (chromosome 10, ORF 76, also known as Armadillo-like helical domain-containing protein 3 [ARMH3]) was identified as a participant of the replication of CV-A10 and PV but not CV-B1 (McPhail et al., 2020; Voilquin et al., 2020). PI4KB can recruit c10orf76 to the c10orf76-dependent virus ROs membrane via the c10orf76-PI4KB interface. c10orf76 on the ROs may contribute to proper Arf1 activation and increase the PI4P level of ROs (McPhail et al., 2020; Table 1; Figure 3).

### Cholesterol

Cholesterol, intercalated between phospholipid bilayers, is an important component of the cell membrane. It is related to membrane fluidity and curvature (Nchoutmboube et al., 2015). The ROs induced by enteroviruses, such as HRV, CVB3, PV, and echovirus, are enriched in cholesterol. Cholesterol can promote enterovirus replication and regulate the process of proteolysis of polyproteins related to virus replication and package. In addition, it can offset the increased fluidity caused by PI4P (Ilnytska et al., 2013; Arita, 2014; Roulin et al., 2014; Albulescu et al., 2015b). In contrast, echovirus 1 replication is not related to cholesterol (Siljamaki et al., 2013). The reason for the above different results may be due to different research strategies.

**FIGURE 4:**
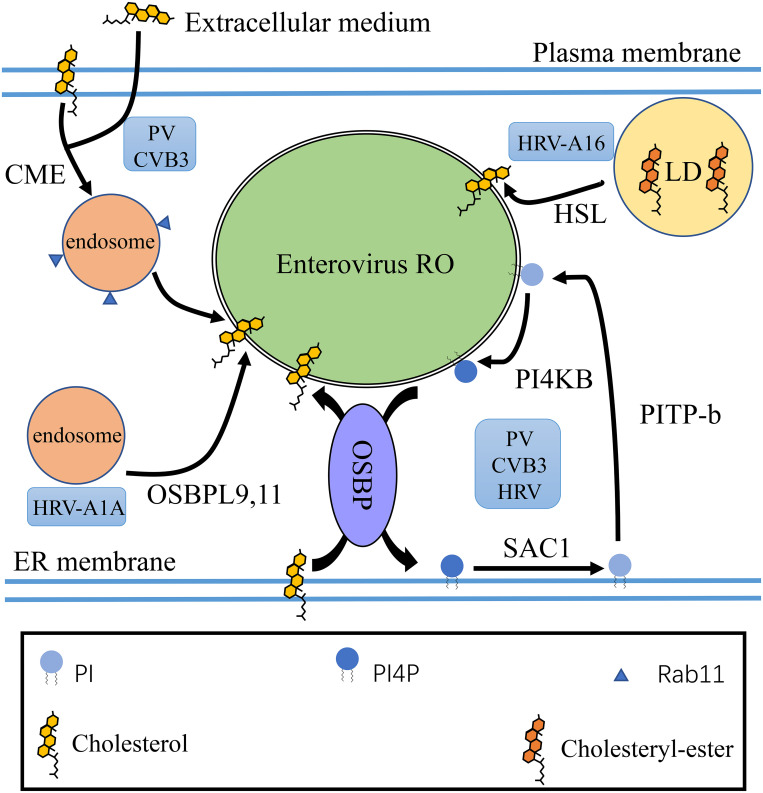
Cholesterol homeostasis of enterovirus ROs. Different enteroviruses utilize multiple mechanisms to enrich cholesterol on ROs. HRV-A16 is dependent on HSL to hydrolyze cholesterol ester stored in LDs. PV and CVB3 activate CME to redistribute cholesterol from the plasma membrane and extracellular medium to ROs and harness Rab11 recycling endosomes to target cholesterol to ROs. Enteroviruses such as PV, CVB3, and HRV can also use OSBP and other host factors driving PI4P-cholesterol countercurrents to increase the level of cholesterol in ROs. OSBP mediates the transfer of PI4P from the ROs to the ER with cholesterol transfer from the ER to the ROs. PI4P is dephosphorylated to PI by Sac1 on the ER membrane. PI is transported to enterovirus ROs from the ER by PITP-b. On the membrane of enterovirus ROs, PI is phosphorylated to PI4P by PI4KB (Roulin et al., 2014; Nchoutmboube et al., 2015).

There are three ways to improve the content of ER membrane cholesterol. One is *de novo* biosynthesis. Cholesterol is produced from acetyl-CoA via the mevalonate pathway, and the rate limiting enzyme 3-hydroxy-3-methylglutaryl-CoA reductase (HMGCR) is involved. The second is uptaking extracellular cholesterol through late endosomes. The third way to generate cholesterol by hydrolyzing cholesteryl-ester in LDs or late endosomes (Ikonen, 2008). Replication of enterovirus is not sensitive to the inhibition of HMGCR, indicating that *de novo* cholesterol synthesis does not participate in enterovirus replication (Roulin et al., 2014; Albulescu et al., 2015b; Luo et al., 2020). HRV-A16 depends on cholesteryl-esterase hormone-sensitive lipase (HSL) to hydrolyze cholesterol-ester stored in LDs, as HRV-A16 is sensitive to HSL inhibitors. However, HRV-A1A relies on OSBP-like (OSBPL) proteins, like OSBPL9 and OSBPL11 (Roulin et al., 2014). During PV and CVB3 infection, the 2BC protein activates clathrin-mediated endocytosis (CME) to redistribute cholesterol from the plasma membrane and extracellular medium. Cholesterol entering the cytoplasm is transported into Rab11 recycling endosomes. The viral 3A protein recruit PI4KB, which directly binds Rab11, to harnesses Rab11 recycling endosomes, which transport cholesterol to ROs and prevent cholesterol back to the plasma membrane (Hsu et al., 2010; Ilnytska et al., 2013; Strating et al., 2013). In addition, HRV, PV, and CVB3 are reported to use OSBP and other host factors driving PI4P-cholesterol countercurrents to increase the level of cholesterol in ROs (Arita, 2014; Roulin et al., 2014). OSBP contain an FFAT motif that bind to VAP; an pleckstrin homology (PH) domain to bind PI4P, through which OSBP can connect many organelles; and the OSBP-related domain (ORD) that binds and transports cholesterol and quickly exchanges cholesterol at membrane contact sites (MCSs) (Mesmin et al., 2017; Ikonen, 2018). After enterovirus infection, OSBP establishes MCSs between the ER and the ROs in order to maintain condition with high cholesterol content in ROs. OSBP mediates the transfer of PI4P from the ROs to the ER while cholesterol transfer from the ER to the ROs (Table 1). Sac1, which converts PI4P to PI on ER membrane, and PI transfer protein beta (PITP-b), which transports PI to the ROs, where PI is phosphorylated by PI4KB to PI4P, are vital for completing the cycle (Mesmin et al., 2013; Roulin et al., 2014; Nchoutmboube et al., 2015; van der Schaar et al., 2016a; Figure 4). Knockdown or pharmacological inhibition of OSBP inhibited the replication of enterovirus (Roulin et al., 2014; Strating et al., 2015). Moreover, after silencing key proteins in PI4P-cholesterol cycle between ER and RO, enterovirus replication were damaged, indicating that the PI4P-cholesterol cycle between ER and RO have important effect on the replication of enterovirus (Hsu et al., 2010; Roulin et al., 2014).

## Inhibitors

### Target PI4KB

PIK93 is the first discovered specific PI4KB inhibitor. It can inhibit PI4KA at a concentration 100 times higher than that of PI4KB, and it can also inhibit phosphatidylinositol 3-kinase (Knight et al., 2006). PIK93 can inhibit PI4KB to inhibit the interaction of viral 3D^pol^ with PI4P on the RO membrane. For the high cross reactivity of PIK93, designing more potent and more selective PI4KB inhibitors is an important field of research (Rutaganira et al., 2016). Enviroxime is found to have potent activity against rhinovirus in 1980 (Wikel et al., 1980). Then it is identified to target PI4KB to against virus (van der Schaar et al., 2012). Clinical studies show that enviroxime can relieve symptoms after virus infection, but the therapeutic activity is disappointing (Phillpotts et al., 1981, 1983; Hayden and Gwaltney, 1982; Levandowski et al., 1982; Miller et al., 1985; Higgins et al., 1988). Most of the compounds that directly inhibit PI4KB activity, as well as enviroxime, are known as major enviroxime-like compounds. MDL-860, an atypical enviroxime-like compound, is a compound with broad-spectrum antiviral activity. It is one of the few drug candidates that has shown effective antiviral infection in *in vivo* experiments. MDL-860 treatment causes PI4KB covalent modification and irreversible inactivation (Arita et al., 2017). In addition, another major enviroxime-like compounds, such as pachypodol (Ro 09-0179), oxoglaucine, GW5074, BF-738735, and T-00127-HEV1, can also inhibit PI4KB to hamper the formation of ROs (Arita et al., 2009, 2011, 2015; MacLeod et al., 2013; van der Schaar et al., 2013; Ford Siltz et al., 2014; Bauer et al., 2017; Table 2).

**TABLE 2:** The inhibitors targeting Enterovirus RO.

Targeted protein	Inhibitor	References
PI4KB	PIK93	Knight et al., 2006; Rutaganira et al., 2016
	enviroxime	van der Schaar et al., 2012
	MDL-860	Arita et al., 2017
	pachypodol	Arita et al., 2015
	oxoglaucine	Arita et al., 2015
	GW5074	Arita et al., 2009; Arita et al., 2011; Ford Siltz et al., 2014
	BF-738735	MacLeod et al., 2013; van der Schaar et al., 2013
	T-00127-HEV1	Arita et al., 2011; MacLeod et al., 2013
OSBP	AN-12-H5	Arita et al., 2013
	T-00127-HEV2	Arita et al., 2013
	25-HC	Arita et al., 2013
	ITZ	Strating et al., 2015
	TTP-8307	Albulescu et al., 2017; Roberts et al., 2019b
	OSW-1	Albulescu et al., 2015a; Roberts et al., 2019a, b
COP I	BFA	Crotty et al., 2004
GBF1	GCA	van der Linden et al., 2010
2C (viral protein)	GnHCl	Laufman et al., 2019
ATGL	Atglistatin	Laufman et al., 2019
HSL	CAY10499	Laufman et al., 2019
/	NITD008	Deng et al., 2014

The antiviral function of PI4KB inhibitors has a broad spectrum, as PI4KB acts as a vital part to replicate vRNA. However, some PI4KB inhibitors have shown potential toxicity and side effects (Lamarche et al., 2012; Spickler et al., 2013). In addition, enterovirus can resistant the inhibition by PI4KB inhibitors by mutations in the viral 3A protein (van der Schaar et al., 2012; Thibaut et al., 2014; Arita, 2016).

### Target OSBP

Minor enviroxime-like compounds, such as AN-12-H5, T-00127-HEV2, 25-HC, and itraconazole (ITZ), are reported to target OSBP to inhibit enterovirus ROs formation (Arita et al., 2013; Strating et al., 2015). ITZ is approved as an antifungal drug, so there are many studies about the *in vivo* effects (Shim and Liu, 2014). However, we know little about the behavior of others in animals or human. In *in vitro* tests, TTP-8307 has shown to inhibit lipid diversion by OSBP, so it is a broad-spectrum enterovirus inhibitor (Albulescu et al., 2017; Roberts et al., 2019b). OSW-1 is an antiproliferative natural product compound, which targets OSBP and ORP4. OSW-1 potently inhibited the replication of EV71, CVA21, HRV-2, and HRV-14 by targeting OSBP (Albulescu et al., 2015a). Cellular OSBP level reduced after low-dose and short-term treated with OSW-1, with little cytotoxicity. Transiently treated cell with OSW-1 resulted in the persistent OSBP reduction that reduced echovirus 2 and CV-A9 replication (Roberts et al., 2019a, b; Table 2).

### Other Targets

A previous study found that the replication of enteroviruses decreased after BFA treatment. However, a single amino acid mutation in the 2C and 3A regions led to PV resistance to BFA (Crotty et al., 2004). Golgicide A (GCA) inhibited the replication of CVB3 by targeting GBF1 (van der Linden et al., 2010). Guanidine hydrochloride (GnHCl) reduced the MCSs between LDs and ROs by inhibiting the 2C protein of PV to disrupt the biogenesis of ROs. Atglistatin, an ATGL inhibitor, caused kinetic delay of PV infectious particles production. And CAY10499, an inhibitor target HSL, had an effect on PV replication (Laufman et al., 2019). The adenosine analog NITD008 also showed effective inhibition to EV71 (Deng et al., 2014; Table 2).

## Conclusion

Researches on enterovirus ROs give us a more comprehensive and in-depth understanding of the enterovirus life cycle. In this review, we summarized the current knowledge of enterovirus ROs, including their function, morphology, biogenesis, and generation mechanism. Enteroviruses share significantly conserved mechanisms to build ROs. The proteins involved in the secretory pathway, the autophagy pathway, and lipid metabolism are all vital for ROs formation. We have found many host factors related to ROs, but several other host proteins that have been proved to play a role in enterovirus replication are often poorly understood. More researches are needed to illuminate how the virus hijacks host factors to initiate the reorganization of the membrane and maintain such an unstable membrane. The current researches on the role of lipids in the formation of ROs are focused on PI4P, and study on other lipids is poor. The function of other lipids remains to be studied. In addition, there are more studies needed to be done to better comprehend the role of ROs in enterovirus life cycle, especially regarding how viruses utilize ROs compartments to consort different steps, such as replication, assembly, and release to achieve enterovirus efficient replication.

## Author Contributions

XL conceived, designed, and wrote the manuscript. MW and AC revised the manuscript. XW, XO, SM, QG, DS, RJ, QY, YW, DZ, XZ, SC, ML, SZ, YL, YY, LZ, BT, LP, and XC helped with the manuscript. All authors read and approved the final manuscript for publication.

## Conflict of Interest

The authors declare that the research was conducted in the absence of any commercial or financial relationships that could be construed as a potential conflict of interest.
